# Contributions of different mosquito species to the transmission of lymphatic filariasis in central Nigeria: implications for monitoring infection by PCR in mosquito pools

**DOI:** 10.1186/1475-2883-6-14

**Published:** 2007-11-29

**Authors:** Audrey Lenhart, Abel Eigege, Alphonsus Kal, D Pam, Emmanuel S Miri, George Gerlong, J Oneyka, Y Sambo, J Danboyi, B Ibrahim, Erica Dahl, D Kumbak, A Dakul, MY Jinadu, John Umaru, Frank O Richards, Tovi Lehmann

**Affiliations:** 1 The Carter Center, Atlanta, GA, USA and Jos, Nigeria; 2 NCID/DPD/Entomology Branch, Centers for Disease Control and Prevention, Atlanta, GA, USA; 3 State Ministry of Health, Nasarawa State, Lafia, Nigeria; 4 Federal Ministry of Health, Lagos, Nigeria; 5 Laboratory of Malaria and Vector Research, NIAID, NIH, Rockville, MD, USA; 6 Department of Zoology, University of Jos, Jos, Nigeria; 7 State Ministry of Health, Abuja, Nigeria

## Abstract

**Background:**

Members of the *Anopheles gambiae *complex are important vectors of lymphatic filariasis (LF) in sub-Saharan Africa, but little is known about the relative contributions of all mosquitoes to lymphatic filariasis transmission in this area.

**Methods:**

Over a 28 month period, mosquitoes were collected from 13 villages in Plateau and Nasarawa states in central Nigeria and dissected to determine *W. bancrofti *infection status. Wings and legs from a subset of the mosquitoes visually identified as A. *gambiae s.l. *were identified by PCR as either *A. gambiae s.s. *or *A. arabiensis*.

**Results:**

*A. gambiae s.s *peaked in abundance during the rainy season while *A. arabiensis *predominated during drier parts of the year. Both species were found equally likely to be infected with the developing stages (L_1_-L_3_) of *W. bancrofti *(9.2% and 11.1%, respectively). Fewer *A. funestus *(1.1%, p < 0.001) were infected than *A. gambiae s.l.*

**Conclusion:**

Understanding the relative contributions of morphologically indistinguishable species to LF transmission is essential if PCR is to be performed on mosquito pools. In the study area, the use of mosquito pools composed of *A. gambiae *sibling species would not be problematic, as both *A. gambiae s.s. *and *A. arabiensis *contribute equally to LF transmission.

## Background

Infection by the filarial parasite, *Wuchereria bancrofti*, is the most common cause of lymphatic filariasis (LF), accounting globally for approximately 90% of all infections. Worldwide, over 120 million people are infected with lymphatic filariasis, with 20% of the global population (over 1.1 billion people) at risk for infection [1]. In Africa, the prevalence of lymphatic filariasis is especially striking, affecting over 40 million people in the sub-Saharan region alone [2]. Overall, Africa is thought to account for 40% of all cases of lymphatic filariasis in the world [3]. In 1997, the World Health Organization passed resolution 50.29, prioritizing "the elimination of lymphatic filariasis as a public health problem." This resolution was adopted due to the nature of the disease as well as the availability of new means to control the transmission of lymphatic filariasis [1], in particular, the use of a strategy of annual combined mass drug administration to reduce microfilaremia, which in turn also reduces vector infection rates. Large-scale transmission interruption programs have been launched in recent years that are based on a strategy of annual mass distribution of the drugs ivermectin (donated through Merck's Mectizan^® ^Donation program) and albendazole (donated by GlaxoSmithKline).

A PCR assay has previously been developed to amplify the *Ssp *I repeat region on the *W. bancrofti *genome, providing a highly sensitive method for detecting *W. bancrofti *DNA in both individual and pooled mosquitoes [4,5]. As lymphatic filariasis control programs progress, PCR will become an increasingly important tool in certifying the absence of *W. bancrofti *infection in vector populations. PCR on pools of mosquitoes, rather than individual mosquitoes, allow a far greater number of mosquitoes to be processed at one time. As transmission rates approach zero after several years of annual mass drug treatment, performing PCR assays on mosquito pools can provide a cost-effective, highly sensitive and specific way to monitor decreasing levels of transmission [6,7]. PCR assays on pooled mosquitoes have been proven to successfully estimate local levels of transmission of lymphatic filariasis expressed in rates of vector infection [6]. In addition, monitoring changes in mosquito infection rates can provide a timely index of transmission dynamics, as changes in infection patterns in humans could take years to detect [8].

Members of the *Anopheles gambiae *complex are important vectors of lymphatic filariasis in sub-Saharan Africa. In central Nigeria, where *W. bancrofti *is the etiological agent, little is known about the mosquitoes that act as vectors. Mosquitoes of the *A. gambiae *(principally *A. gambiae s.s. *and *A. arabiensis*) and *Anopheles funestus *complexes are known to be involved [9], but nothing is known about the relative contributions of *A. gambiae s.s.*, *A. arabiensis*, and *A. funestus *to LF transmission. Previous studies of the *A. gambiae *complex in Kaduna in northern Nigeria have shown that *A. gambiae s.s *predominates in the cooler, wet season while *A. arabiensis *predominates in the hotter, drier season [10]. Understanding the differences in the likelihood of infection between species and their ability to support developing parasites is important to understanding spatial and temporal variations in transmission intensity and in monitoring control efforts. Such knowledge is a prerequisite for applying PCR assays to pools of mosquitoes that may include a mix of sibling species. For pooling, *A. funestus *is easily distinguished visually and separated from *A. gambiae s.l.*, but the sibling species *A. gambiae s.s. *and *A. arabiensis *are morphologically indistinguishable and would be combined in pools when monitoring LF transmission by PCR in an operational setting. A PCR assay has previously been developed to distinguish between these two sibling species [11].

We discuss our findings using dissection and PCR to describe the relative abundance and *W. bancrofti *infections found in the three LF vectors in an endemic region in central Nigeria, and to evaluate if pooling different vector species for *W. bancrofti *detection by PCR might confound results and mislead data interpretation.

## Methods

### Study Area

The study area included 13 villages in Plateau and Nasarawa States in the Jos plateau of central Nigeria. The average elevation was 4200 feet (1300 m), with average monthly temperatures ranging from a low of 76°F (24°C) in July and August to a high of 87°F (30°C) in March. Average monthly precipitation ranges from a low of 0 inches per month from November through February and peaks at 12 inches (304 mm) in July.

### Mosquito Collection

Early morning live mosquito collections by aspiration took place in housing compounds over a 28 month period from August of 1999 to November 2001. Live mosquitoes were stored in paper cups and provided with sugar water until dissection (up to six hours after collection). A visual identification of species was made, categorizing mosquitoes as either *A. gambiae s.l.*, *A. funestus*, *Culex *species, *Aedes *species or 'other.' Mosquitoes were dissected individually to determine *W. bancrofti *infection status, including stage and location of the parasites in the mosquito body. Microfilaria (mf) stages were also counted, but not included in the calculation of infection rates. Ovaries were spread and dried for parity determination and the abdomen, thorax and head were placed in separate drops of saline water and dissected to count all stages of *W. bancrofti *larvae. Legs and wings were removed and desiccated for future PCR species analysis. Due to collection and transportation complications, only a subset of legs and wings from dissected individuals were ultimately available for species PCR analysis.

### PCR Protocol

To differentiate between the species of mosquitoes visually identified as *A. gambiae s.l*., DNA was extracted using the method described by Collins *et al. *[12]. Species diagnostic primers were manufactured to amplify the 28S coding region of the ribosomal DNA intergenetic spacer [11]. The total volume of the PCR reaction was 25 μl: 18.663 μl sterile deionized H_2_O, 2.5 μl Promega^® ^10 × buffer, 0.312 μl of GA (20 ng/μl), 0.625 μl of UN (20 ng/μl), 0.925 μl of AR (20 ng/μl), 0.5 μl of 2.5 mM dNTP mix, 0.3 μl of 25 mM MgCl_2_, and 0.175 μl of Promega^® ^Taq Polymerase (5 units/μl). A 1.0 μl aliquot of the stock DNA extract was used as the DNA template. Positive and negative controls for the PCR were run with each reaction. The positive controls consisted of samples containing *A. gambiae s.s. *and *A. arabiensis *DNA, and sterile, deionized water was used as a negative control. The temperature program for the PCR reaction was: 94°C for 5 minutes, 30 cycles (94°C for 30 seconds, 55°C for 30 seconds, 72°C for 30 seconds), and a final extension of 72°C for 5 minutes. The PCR product was visualized on a 2% agarose gel stained with ethidium bromide alongside a 100 base pair ladder.

### Data Analysis

Data were analysed using SAS version 8 and Epi Info software.

## Results

Of 4898 mosquitoes dissected, 143 (2.9%) contained developing stages (L_1_-L_3_) of *W. bancrofti *larvae. *A. gambiae s.l. *comprised 89.5% (128/143) of the positive mosquitoes and *A. funestus *accounted for the other 10.5% (15/143). A significantly greater proportion of *A. gambiae s.l. *(3.7%, 128/3473) were infected with developing parasites as compared to *A. funestus *(1.1%, 15/1352) (p < 0.001, RR = 3.41). In addition, 84 *A. gambiae s.l. *(2.4%) were found to contain microfilariae, along with 14 *A. funestus *(1.0%) and 2 *Culex spp*. (2.8%) (Table 1). *A. gambiae s.l. *was significantly more likely to contain microfilariae than *A. funestus *(p = 0.003, RR = 2.34), but not *Culex*.

**Table 1:** Summary table of mosquito species and infection status.

	***A. gambiae s.l.***	**Analysed by PCR [n]**			***A. funestus***	***Culex spp.***	***Aedes spp.***	**Total**
	
		*A. gambiae s.s.*^a^	*A. arabiensis*^a^	Unidentified^b^				
						
**Dissected N**	3473	[587]	[108]	[18]	1352	71	2	4898
**Mff + n (%)**	84 (2.4)	[17 (2.9)]	[1 (0.9)]	[0]	14 (1.0)	2 (2.8)	0	100 (2.0)
**L_1_-L_3 _+ n (%)**	128 (3.7)	[54 (9.2)]	[12 (11.1)]	[4 (22.2)]	15 (1.1)	0	0	143 (2.9)
**L_3 _+ n (%)**	49 (1.4)	[27 (4.6)]	[6 (5.6)]	[3 (16.7)]	7 (0.5)	0	0	56 (1.1)

^a ^Species confirmed by PCR

^b^Species by PCR neither *A. gambiae s.s. *nor *A. arabiensis*; presumably misidentified *A. funestus*

The legs and wings of 713 of the dissected *A. gambiae s.l. *individuals underwent PCR assays for species identification. The PCR results showed that 587 (82.3%) of these individuals were *A. gambiae s.s.*, 108 (15.2%) were *A. arabiensis *and 18 (2.5%) did not produce any PCR product. The specimens that were neither *A. gambiae s.s*. nor *A. arabiensis *by PCR were presumed to be visually misidentified and were most likely *A. funestus*.

A total of 70 (9.8%) of the 713 *A. gambiae s.l. *individuals that underwent PCR analysis for species identification had been determined by dissection to be infected with L_1_-L_3_. Of these, 77.1% were determined by PCR to be *A. gambiae s.s. *(54/70), 17.1% were *A. arabiensis *(12/70), and the remaining 5.7% (4/70) were neither (Table 1). Although the proportion of *A. arabiensis *positive for developing stages of the parasite (11.1%, 12/108) was slightly greater than *A. gambiae s.s. *(9.2%, 54/587), this difference was not significant (p > 0.05). The proportions of *A. arabiensis *and *A. gambiae s.s. *positive for stage L_3 _infective larvae and microfilariae also did not differ.

**Figure 1:**
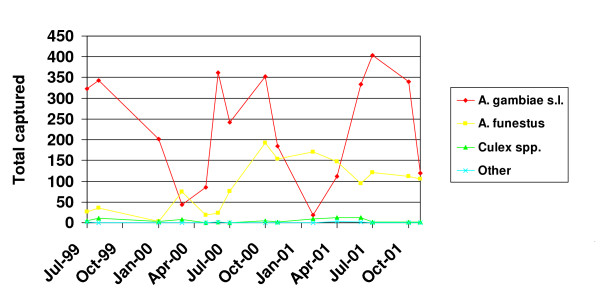
Seasonal variations in the abundance of mosquitoes captured throughout the study period.

**Figure 2:**
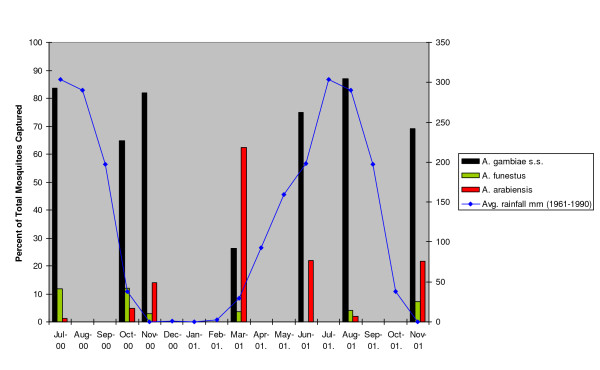
Seasonal variations in the relative proportions of *A. gambiae s.s*, *A. arabiensis *and *A. funestus *in the village of Lankan.

**Figure 3:**
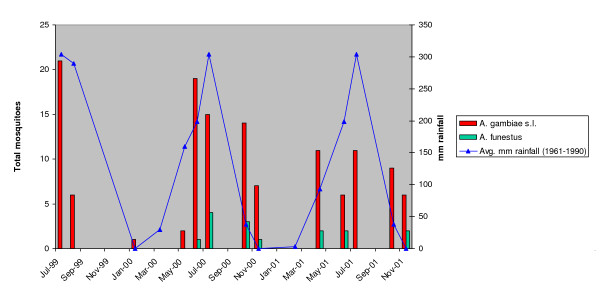
Seasonal variations in the number of mosquitoes found positive by dissection for developing stages of the *W. bancrofti *parasite, plotted together with rainfall data.

Abundance of all mosquitoes dropped during the dry season, during which time *A. funestus *was found in greater numbers than *A. gambiae s.l. *(Figure 1). The relative abundance of *A. gambiae s.s. *and *A. arabiensis *over time in relation to rainfall for the village of Lankan (located within the study area) is shown in Figure 2. Around the peaks of the rains, *A. gambiae s.s. *was most abundant, but as the rains began and ended, *A. arabiensis *became most abundant. Despite these seasonal fluctuations in vector abundance, mosquitoes positive for infective *W. bancrofti *parasites were found throughout the year, indicating that LF transmission occurs year-round in this area. However, variations in the number of mosquitoes found with developing larvae followed rainfall patterns (Figure 3), showing that transmission was likely highest during the time of year when *A. gambiae s.s. *was most abundant, coinciding with the peak of the rainy season.

## Discussion and Conclusion

The results presented here describe the relative contributions of *A. gambiae s.s.*, *A. arabiensis *and *A. funestus *to LF transmission in central Nigeria. *A. funestus *was shown to be less likely to harbor developing stages of larvae than *A. gambiae s.l. *The sibling species of the *A. gambiae *complex were proportionally equally likely to carry infective stages of the *W. bancrofti *parasite, although they experienced different seasonal fluctuations in abundance. No developing parasites were found in any of the *Culex *mosquitoes dissected.

These variations in vectorial capacity are especially important when considering pooling mosquitoes for PCR analysis. *A. funestus *is morphologically distinguishable from *A. gambiae s.l*., making its potential use in mosquito pools less problematic. However, *A. gambiae s.s. *and *A. arabiensis *are distinguishable only by molecular analysis. From the data presented here, it does not appear that pooling *A. gambiae s.s. *and *A. arabiensis *would result in inappropriate conclusions regarding transmission dynamics in central Nigeria. While in this area *A. gambiae s.s. *and *A. arabiensis *appear to have the same vectorial capacity, in other places this may not be the case, and pooling them together for PCR analysis could cause problems if one is a more important vector than the other.

As lymphatic filariasis programs move toward elimination, the use of PCR to identify *W. bancrofti *infection in mosquitoes should become an increasingly important tool in certifying the absence of LF transmission. However, important limitations exist when linking the results from PCR assays of vector mosquitoes to LF transmission. The current *W. bancrofti *PCR assay is not stage-specific, so it is impossible to know if a positive mosquito pool contains the infective L_3 _stage of the parasite. Indeed, the sensitivity of the assay is such that a positive result could potentially be achieved with only the remnants of dead parasites present in a mosquito, as has recently been shown in PCR assays of mosquitoes for *Brugia malayi *[13].

Overall, PCR becomes more cost effective than dissection when mosquito infection levels are low and when pools of mosquitoes can be tested. As infection levels drop, it becomes increasingly difficult and more costly and time consuming to dissect large numbers of mosquitoes with little or no infection ever detected [8]. However, before the mosquitoes of a region can be pooled for analysis, preliminary studies such as the one reported here should be performed on individual mosquitoes, particularly if several different species are vectors, to assess the relative contribution of each species to transmission. Different regions contain unique sets of characteristics that influence LF transmission, and the application of molecular and entomological indices should be used accordingly. In our study, we found that morphologically indistinguishable sibling species provide similar contributions to LF transmission, allowing us to visually separate our pools for molecular testing. Operationally, this finding is critical to allow entomological indices to be correctly measured and proper conclusions drawn.

## Competing interests

The author(s) declare that they have no competing interests.

## Authors' contributions

AL carried out the PCR assays, data analysis and drafted the manuscript. TL designed the field study and oversaw the dissections together with AE and AK. DP, ESM, GG, JO, YS, JD, BI, DK, AD, MYJ and JU all participated in the fieldwork. ED participated in the PCR assays. FR and TL conceived of the study, and participated in its design and coordination and helped to draft the manuscript.
